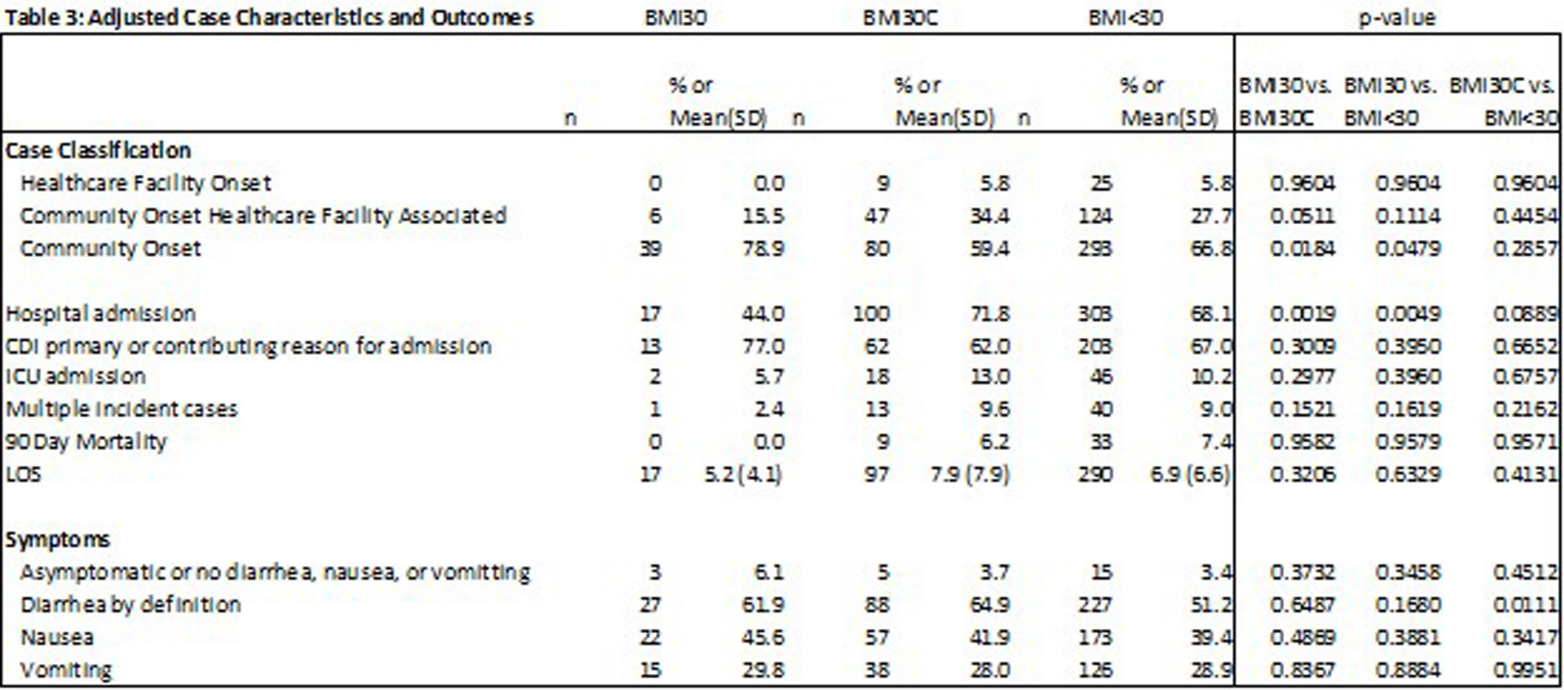# 129 Examining De-escalation of Anti-pseudomonal Antibiotics in Hospitalized Patients After a Microbiology Reporting Nudge

**DOI:** 10.1017/ash.2026.10541

**Published:** 2026-06-23

**Authors:** Sarah Petersen, Natalie Stanley, Samantha Mathieson, Malakai Miller, Mary Beth Blair

**Affiliations:** 1 Tennessee Department of Health; 2 TN Department of Health

## Abstract

**Background:** Clostridioides difficile infection (CDI) is a common healthcare-associated infection. Despite its well-characterized nature, literature remains divided on the impact of obesity on CDI. Through the Emerging Infections Program’s CDI surveillance, we investigated the influence of obesity — with and without comorbidities, on CDI compared to non-obese patients residing in Davidson County, Tennessee. **Methods:** Fully abstracted CDI cases, defined as the first positive stool test for persons at least 1 year old, residing in Davidson County, Tennessee from 2016–2023, were examined. Study groups included BMI ≥ 30 with no comorbidities (BMI30), BMI ≥ 30 with at least 1 comorbidity (BMI30C), and BMI < 30 (BMI<30). Pairwise comparisons of characteristics and outcomes were made via logistic regression custom hypothesis tests for categorical variables and Tukey-Kramer test for continuous variables. All outcomes were adjusted for age; stepwise logistic regression determined significantly associated patient characteristics for further adjustments. Statistics were conducted using SAS 9.4. **Results:** There were 623 patients identified (BMI30: n=45, BMI30C: n=136, BMI<30: n=442). BMI30 had the highest proportion of female patients (BMI30: 77.8%, BMI30C: 61.0%, BMI<30: 59.5%) and were the youngest group (BMI30: 47.0 years, BMI30C: 57.8 years, BMI<30: 59.0 years). BMI30C were more likely to be Black than BMI<30 (BMI30C: 36.0%, BMI<30: 16.7%). BMI30C were more likely than BMI<30 to be diagnosed with diabetes, chronic kidney disease, neuropathy, or a chronic ulcer/wound of the skin. BMI30 were the most likely to be community-onset (adjusted: BMI30: 78.9%, BMI30C: 59.4%, BMI<30: 66.8%) and least likely to be community-onset healthcare facility associated (adjusted: BMI30: 15.5%, BMI30C: 34.4%, BMI<30: 27.7%). BMI30C were more likely to have diarrhea (≥3 unformed stools in one day) than BMI<30 (BMI30C: 64.9%, BMI<30: 51.2). BMI30 were least likely to be admitted (adjusted: BMI30: 44.0%, BMI30C: 71.8%, BMI<30: 68.1%). Among admitted patients, there were no significant differences in ICU admissions or length of stay. There were no significant differences in 90-day mortality, number of cases, proton pump inhibitor, H2 blocker, or antibiotic use in any groups. Detailed statistics are available in tables 1–3. **Conclusion:** Variances in symptoms, case classification, and admissions, paired with similarities in mortality and length of stay between groups, demonstrate that providers should consider a patient’s unique health history when managing CDI, rather than adopting a uniform approach for all obese patients. Future studies with more diverse patient populations could guide clinicians in bettering the treatment and prevention of CDI in at-risk populations.

**Figure:**
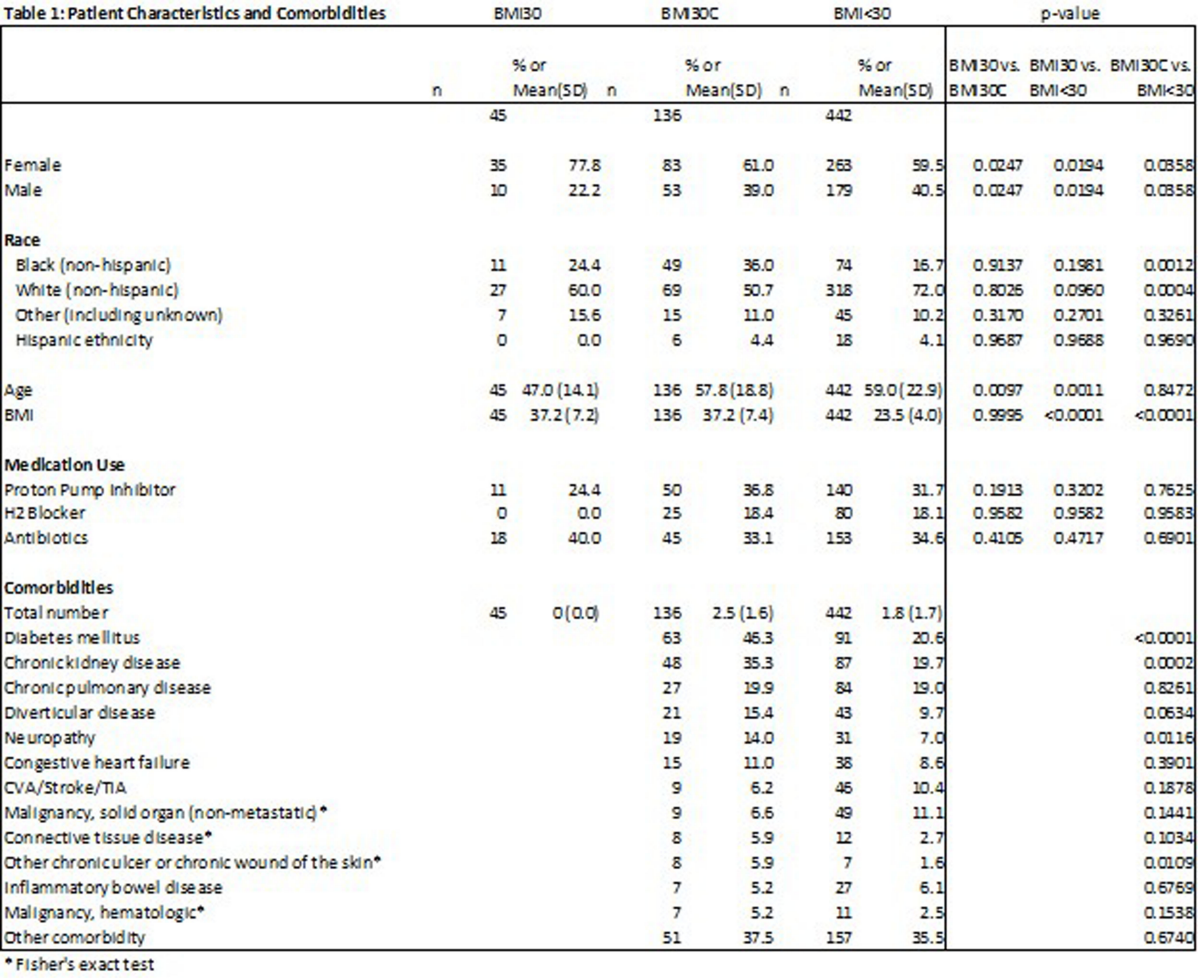

**Figure:**
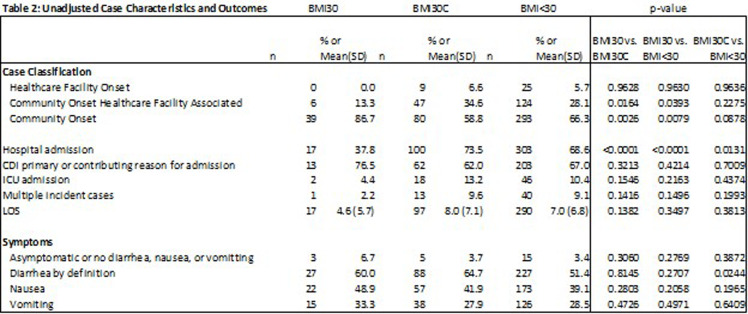

**Figure:**